# Piezosurgery in periodontology and oral implantology

**DOI:** 10.4103/0972-124X.60229

**Published:** 2009

**Authors:** Hema Seshan, Kranti Konuganti, Sameer Zope

**Affiliations:** *Department of Periodontics, M.S. Ramaiah Dental College and Hospital, Bangalore - 560 054, India*

**Keywords:** Osteotomy, piezosurgery, sinus grafting, ultrasonic device

## Abstract

Periodontitis is a chronic inflammatory disease of the tooth-supporting structures. The treatment of this condition is largely based on the removal of local factors and restoration of the bony architecture. Moreover, in the era of modern dentistry, successful implant therapy often requires sound osseous support. Traditionally, osseous surgery has been performed by either manual or motor-driven instruments. However, both these methods have their own advantages and disadvantages. Recently, a novel surgical approach using piezoelectric device has been introduced in the field of periodontology and oral implantology. This article discusses about the wide range of application of this novel technique in periodontology.

## INTRODUCTION

Dental surgical techniques have been developed rapidly over the last two decades. In this article, one new surgical technique based on the novel application of the principle of piezoelectric ultrasonic vibration is introduced with wide range of applications in dentistry and periodontics.

Traditionally, osseous surgery has been performed by either manual or motor-driven instruments. Manual instruments offer good control when used to remove small amounts of bone in areas with relatively less dense mineralization. However, manual instruments are difficult to control in cortical bone, particularly where precise osteotomies are essential. As a consequence, they are mostly applied for gross cutting of larger bone segments. Motor-driven instruments are often used when bone is very dense. Motor-driven instruments transform electric or pneumatic energy into mechanical cutting action using the sharpened edge of burs or saw blades. These instruments generate a significant amount of heat in the cutting zone that must be minimized by water irrigation. Overheating of adjacent tissue may alter or delay the healing response.[1] Reduced rotational speed de-creases not only frictional heat but also cutting efficiency.

Motorized cutting tools also decrease tactile sensitivity. Slower rotational speed necessitates increased manual pressure, which increases the macrovibration of the cutting tool and further diminishes sensitivity. This is particularly troublesome when cutting across an area of dense cortical bone into either trabecular bone or soft tissue, as when drilling an osteotomy above the mandibular canal or preparing a lateral window for sinus grafting. The applied force necessary to cut through the denser bone must be instantaneously released when encountering the less dense tissue or the underlying structures may be damaged.

## WHAT IS PIEZOSURGERY?

**Figure 1:**
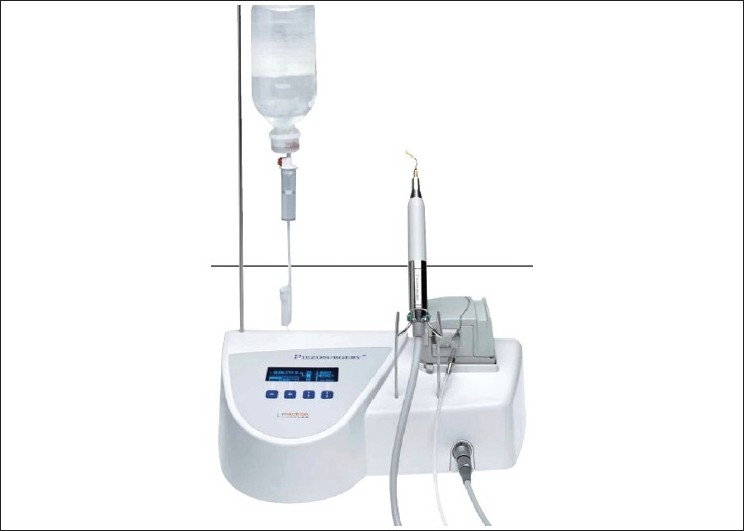
Piezosurgery unit

**Figure 2:**
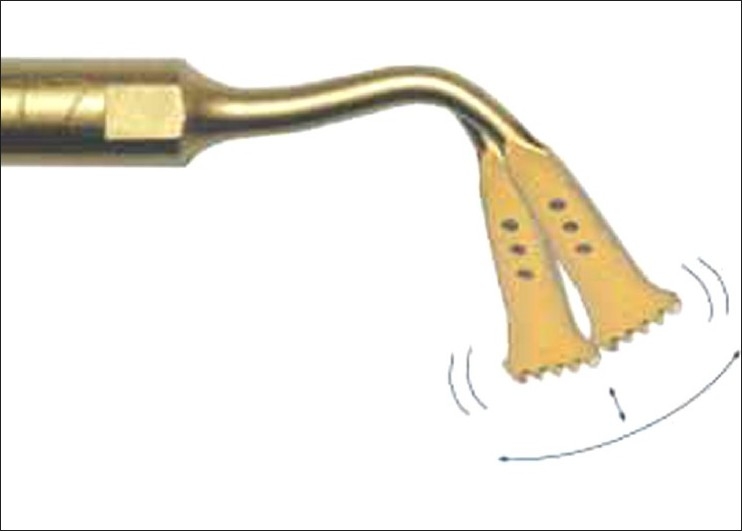
Piezosurgery tip vibrations

Piezosurgery is a relatively new technique for osteotomy and osteoplasty that utilizes ultrasonic vibration. The piezosurgery device is essentially an ultrasound machine with modulated frequency and a controlled tip vibration range [Figure 1]. The ultrasonic frequency is modulated from 10, 30, and 60 cycles/s (Hz) to 29 kHz. The low frequency enables cutting of mineralized structures, not soft tissue. Power can be adjusted from 2.8 to 16 W, with preset power settings for various types of bone density.[2] The piezosurgery tip vibrates within a range of 60-200 mm, which allows clean cutting with precise incisions [Figure 2].

## APPLICATION IN DENTISTRY

Piezosurgical equipment can be used for retrograde preparation of root canal; it performs bone cutting with great precision facilitating ridge augmentation and ridge expansion,[3] tooth extraction, ankylotic tooth extraction[2] and surgical orthodontic surgeries.[4,5]

## APPLICATION IN PERIODONTOLOGY AND IMPLANTOLOGY

The removal of supra and subgingival calculus deposits and stains from teeth, periodontal pocket lavage with simultaneous ultrasonic tip movement, scaling, root planning and crown lengthening, periodontal ostectomy and osteoplasty procedures requires careful removal of small quantities of bone adjacent to exposed root surfaces to avoid damaging the tooth surface.[6] The piezosurgery device is used to develop positive, physiologic architecture of bone support of the involved teeth.

The piezosurgery device can be used for soft-tissue debridement to remove the secondary flap after incision through retained periosteum. By changing to a thin, tapered tip and altering the power setting, the piezosurgery device can be used to debride the field of residual soft tissue and for root surface scaling to ensure thorough removal of calculus.

Osteoplasty and ostectomy is performed using the piezosurgery device to create positive architecture for pocket elimination surgery.[6] The device allows for precise removal of bone, with minimal risk of injury to underlying root surfaces. Final smoothing of root surfaces and bony margins using a specific ultrasonic insert, PP1, creates a clean field, with ideal bony architecture ready for flap closure. The piezosurgery device is used in bone grafting of an infrabony periodontal defect. Autogenous bone can be readily harvested from adjacent sites with minimal trauma and therefore minimal postoperative effects.[1] Implant site preparation, implant removal[7] and bone harvesting, bone grafting and sinus lifts can be done with much ease and less soft tissue trauma.

## ADVANTAGES OF PIEZOSURGERY DEVICE OVER CONVENTIONAL SURGICAL EQUIPMENTS

Compared with traditional rotary instrumentation, piezosurgery requires much less hand pressure. This results in enhanced operator sensitivity and control, indicating that the clinician can develop a better ‘feel’ and precision for the cutting action because of microvibration of cutting tip. The cut is safe because the ultrasonic frequency used does not cut soft tissue. The cutting action is less invasive, producing less collateral tissue damage, which results in better healing.[8,9] Owing to its cavitation effect on physiological solutions (for example, blood), piezosurgery creates a virtually bloodless surgical site that makes visibility in the working area much clearer than with conventional bone cutting instruments. Unlike conventional burs and micro saws, piezosurgery inserts do not become hot, which again reduces the risk of postoperative necrosis.[1]

## CONCLUSION

The piezosurgery device is a new instrument that can be used for bone surgery in a variety of dental surgical specialties.

The advantage of piezosurgery is that it can precisely cut hard tissue, while precluding injury to soft tissue. Minimal heat is generated during cutting, thus maintaining vitality of adjacent tissue. It provides substantial improvement in dental/implant surgery, benefiting the surgeon by ease of use and the patient by minimizing surgical trauma and promoting rapid healing.
